# Exacerbated Imiquimod-Induced Psoriasis-Like Skin Inflammation in IRF5-Deficient Mice

**DOI:** 10.3390/ijms21103681

**Published:** 2020-05-23

**Authors:** Momoko Nakao, Tomomitsu Miyagaki, Makoto Sugaya, Shinichi Sato

**Affiliations:** 1 Department of Dermatology, the University of Tokyo Graduate School of Medicine, Tokyo 113-8655, Japan; orinomomoco@gmail.com (M.N.); sugayamder@gmail.com (M.S.); satos-der@h.u-tokyo.ac.jp (S.S.); 2 Department of Dermatology, St. Marianna University School of Medicine, Kanagawa 216-8511, Japan; 3 Department of Dermatology, International University of Health and Welfare, Chiba 286-0124, Japan

**Keywords:** interferon regulatory factor 5, psoriasis, imiquimod, IL-23, dendritic cells, interferon regulatory factor 4

## Abstract

Interferon regulatory factors (IRFs) play diverse roles in the regulation of the innate and adaptive immune responses in various diseases. In psoriasis, IRF2 is known to be involved in pathogenesis, while studies on other IRFs are limited. In this study, we investigated the role of IRF5 in psoriasis using imiquimod-induced psoriasis-like dermatitis. Although IRF5 is known to play a critical role in the induction of proinflammatory cytokines by immune cells, such as dendritic cells (DCs), macrophages, and monocytes, IRF5 deficiency unexpectedly exacerbated psoriasiform skin inflammation. The interferon-α and tumor necrosis factor-α mRNA expression levels were decreased, while levels of Th17 cytokines including IL-17, IL-22, and IL-23 were increased in IRF5-deficient mice. Furthermore, IL-23 expression in DCs from IRF5-deficient mice was upregulated both in steady state and after toll-like receptor 7/8 agonist stimulation. Importantly, the expression of IRF4, which is also important for the IL-23 production in DCs, was augmented in DCs from IRF5-deficient mice. Taken together, our results suggest that IRF5 deficiency induces the upregulation of IRF4 in DCs followed by augmented IL-23 production, resulting in the amplification of Th17 responses and the exacerbation of imiquimod-induced psoriasis-like skin inflammation. The regulation of IRF4 or IRF5 expression may be a novel therapeutic approach to psoriasis.

## 1. Introduction

Psoriasis is a dendritic-cell (DC) and T-cell-mediated immunologic skin disease with a complex pathogenesis where both genetic and environmental factors are involved [1,2,3,4]. Crosstalk between the innate and adaptive immune system mediated by various cytokines such as interferon (IFN)-α, tumor necrosis factor (TNF)-α and IL-23, is thought to play an essential part in the onset or exacerbation of psoriasis [4,5,6]. In addition, Th17 cells, whose maintenance and activation are driven by IL-23, also fulfill a critical role in the pathogenesis of psoriasis [2]. Moreover, these cytokines also contribute to bone damage, leading to psoriatic arthritis [7]. Actually, blocking antibodies against TNF-α, IL-23p19 and IL-17 show remarkable efficacy in psoriasis and/or psoriatic arthritis [8,9,10]. Although there are several good treatment options, it is still important to elucidate the molecular mechanisms underlying psoriasis in detail, because not all patients can receive benefits from such biologics [11].

Recently, much attention has been focused on the involvement of interferon regulatory factors (IRFs), which were identified primarily as factors regulating the type I IFN system and have been known to have diverse functions in the regulation of the innate and adaptive immune responses in various diseases [12]. Concerning psoriasis, IRF1 and IRF2 are the most studied IRFs. IRF1 has the capacity to mediate the transcriptional activation of type I IFNs, while IRF2 represses the function of IRF1 by competitively binding the same regulatory sequences [13]. At first, immunohistochemical studies revealed altered IRF1 and IRF2 expression in epidermal keratinocytes in psoriatic skin [14,15]. After that, genetically, the variation at the IRF2 gene but not IRF1 gene was found to be associated with susceptibility to psoriasis [16,17]. Moreover, IRF2-deficient mice spontaneously display an inflammatory skin disease similar to human psoriasis because CD8^+^ T cells from IRF2-deficient mice show hyper-responsiveness to type I IFNs [18]. Imiquimod-induced psoriasis-like skin inflammation was severer in IRF-2 hetero-knockout mice than in wild-type mice [19]. These reports suggested that IRF2 is deeply involved in the pathogenesis of psoriasis. On the other hand, the analyses of other IRFs in psoriasis are limited. IRF5 is activated via the Toll-like receptor (TLR)-myeloid differentiation factor 88 (MyD88) pathway in DCs and macrophages and associated with the gene expression of various cytokines [20]. Interestingly, IRF5 gene polymorphisms are linked to the development of various immune and inflammatory disorders including systemic lupus erythematosus, rheumatoid arthritis, multiple sclerosis, inflammatory bowel diseases, myeloperoxidase anti-neutrophil cytoplasmic antibody-associated vasculitis and asthma [21,22,23,24]. In psoriasis patients, there is an interaction between the IRF5 gene variants and major histocompatibility complex (MHC) class I gene locus [25], suggesting that IRF5 should be related to the development of psoriasis. We therefore studied the role of IRF5 in the development of psoriasis using a psoriasis-like inflammation model induced by imiquimod, a ligand for Toll-like receptor (TLR)-7 in IRF5-deficient mice.

## 2. Results

### 2.1. IRF5 Deficiency Exacerbates Psoriasis-Like Skin Inflammation Induced by Imiquimod Treatment

**Figure 1:**
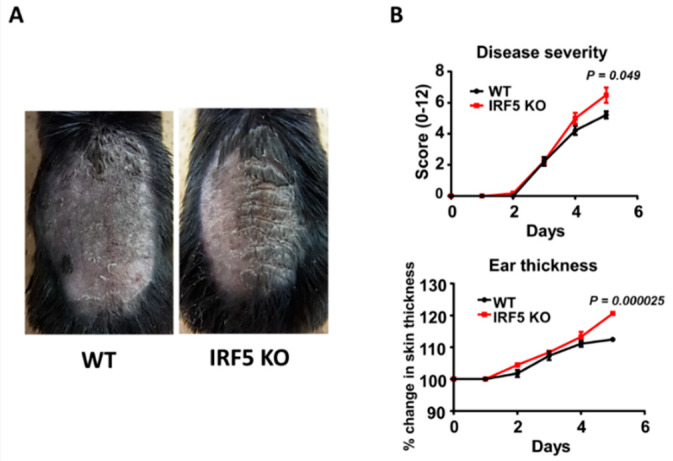
Interferon regulatory factor (IRF)5 deficiency exacerbates psoriasiform dermatitis induced by imiquimod treatment. Shaved back skin and ears of wild-type (WT) and IRF5 knockout (IRF5 KO) mice were topically treated with imiquimod for 5 consecutive days. (**A**) Phenotypical manifestation of WT and IRF5 KO mouse back skin induced by imiquimod application at day 5. Representative photos from 9 mice per group. (**B**) Disease severity and ear thickness during imiquimod treatment. Clinical scores for disease severity were calculated daily using a scoring system based on the clinical Psoriasis Area and Severity Index. Data are presented as the mean ± SEM of three independent experiments (*n* = 9 for each group).

**Figure 2:**
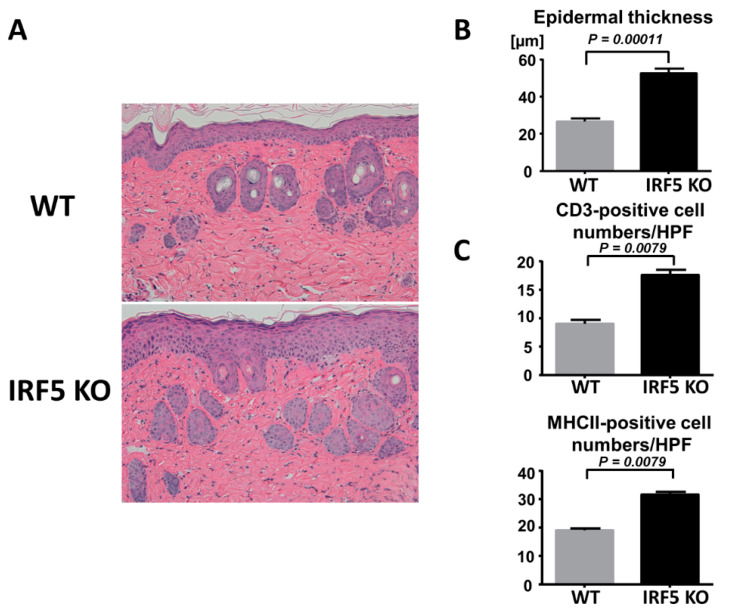
Epidermal hyperplasia and inflammatory cell infiltration are enhanced in IRF5-deficient mice. (**A**) Histological presentation stained with hematoxylin and eosin (HE) of wild-type (WT) and IRF5 knockout (IRF5 KO) mouse back skin induced by imiquimod application at day 5 (×200) Representative pictures from 9 mice per group. (**B**) Epidermal thickness was measured. (**C**) The numbers of CD3^+^ cells and major histocompatibility complex class II (MHCII)-positive cells were counted per high-power field. Data are presented as the mean ± SEM of three independent experiments (*n* = 9).

To examine the role of IRF5 during psoriasis-like skin inflammation, we first applied imiquimod cream on mouse back skin and ear, and investigated the clinical and histopathological features of wild-type (WT) and IRF5 knockout (IRF5 KO) mice. Worse dermatitis with more severe scales was detected in IRF5 KO mice by imiquimod application compared to the WT mice (Figure 1A). Significant differences in disease severity and ear thickness were observed between the WT and IRF5 KO mice at day 5 (Figure 1B). Consistently, the histological examination of back skin samples at day 5 showed severer epidermal hyperplasia and a more intense infiltration of inflammatory cells in IRF5 KO mice than in WT mice (Figure 2A,B). Immunohistochemical analysis revealed that the number of CD3^+^ T cells or major MHC class II^+^ cells in the epidermis and the upper dermis was significantly increased in the IRF5 KO mice compared with the WT mice (Figure 2C). Thus, IRF5 deficiency exacerbated imiquimod-induced psoriasis-like skin inflammation.

### 2.2. IRF5 Deficiency Increases Th17 Cytokine mRNA Levels and Decreases IL-10 mRNA Levels

**Figure 3:**
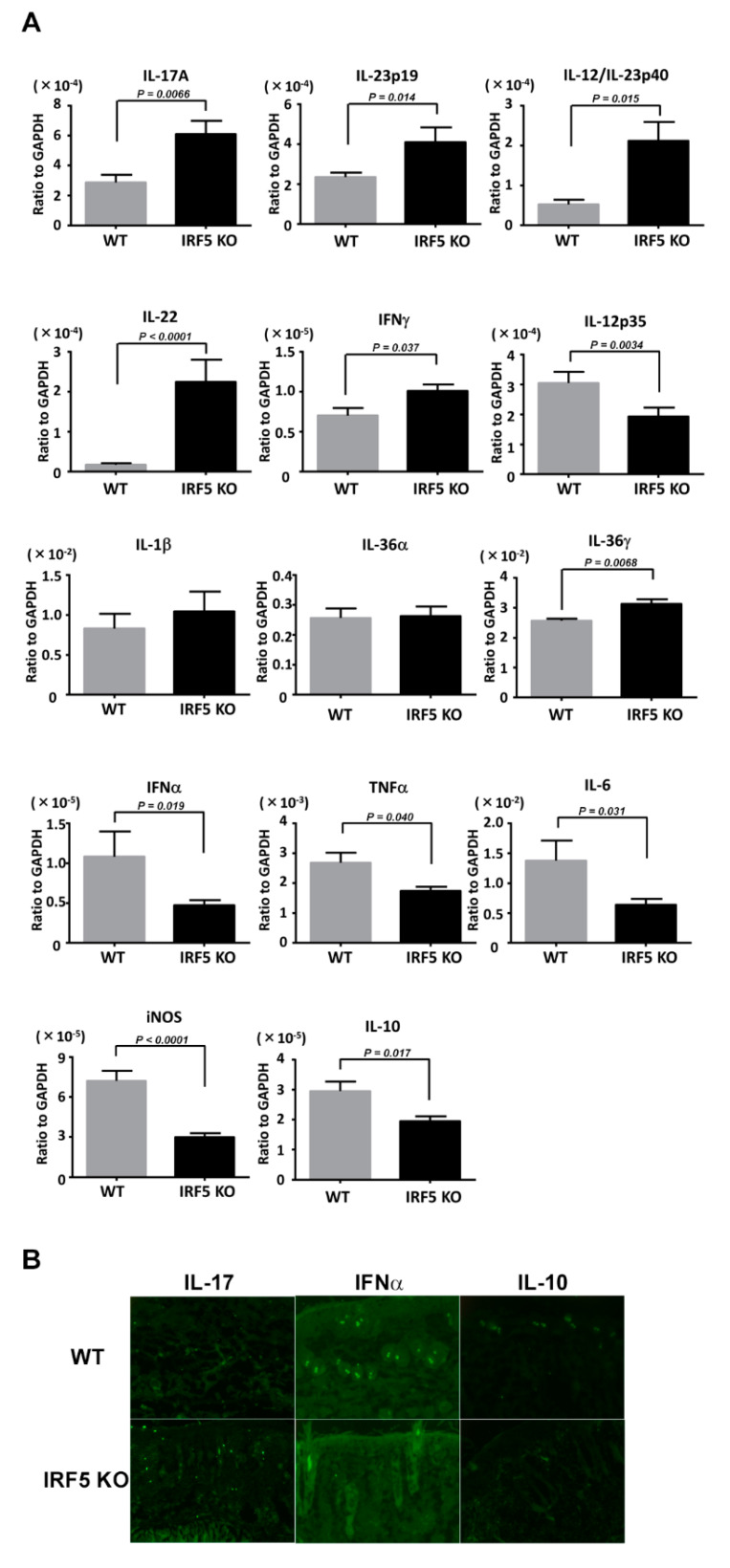
Th17 cytokine expression levels were upregulated and the IL-10 levels were downregulated in the IRF5-deficient mice. (**A**) Wild-type (WT) and IRF5 knockout (IRF5 KO) mice were applied with imiquimod and skin samples were taken 48 h after imiquimod application. Messenger RNA levels of the indicated cytokines were determined by quantitative RT-PCR. Data are obtained from duplicate samples from 12 mice in each group. Values are presented as the mean ± SEM of three independent experiments. (**B**) Immunofluorescent staining of IL-17A, IFN-α and IL-10 of wild-type (WT) and IRF5 knockout (IRF5 KO) mouse back skin induced by imiquimod application at day 5 (×240). Representative pictures from 5 mice per group.

We next examined the messenger RNA (mRNA) expression levels of psoriasis-related cytokines in the skin lesions in WT and IRF5 KO mice. Samples were taken from mouse back skin 48 h after imiquimod application. Interestingly, mRNA levels of Th17 cytokines, such as IL-17A, IL-23p19, IL-23p40 and IL-22 were significantly increased in IRF5 KO mice (Figure 3A). There were no major differences in the mRNA levels for Th1 cytokines, such as IFN-γ and IL-12p35, and IL-1 cytokine family members including IL-1β, IL-36α and IL-36γ between the IRF5 KO mice and the WT mice, although some of them were statistically significantly increased or decreased in IRF5 KO mice (Figure 3A). On the other hand, the mRNA levels of IFN-α, TNF-α, IL-6 and inducible nitric oxide synthase (iNOS) were significantly decreased in the IRF5 KO mice compared with the WT mice (Figure 3A). In addition, the mRNA levels of IL-10 were also significantly diminished in the IRF5 KO mice (Figure 3A). The results are summarized in Table 1. Thus, during the clinical course of imiquimod-induced psoriasis-like skin inflammation, the amplification of Th17 responses caused by the up-regulation of IL-23 expression, the impairment in type I interferon and inflammatory cytokines and the decrease in IL-10 expression occurred in IRF5 KO mice. We also found that the IL-17A^+^ cells were increased and that IFN-α^+^ cells and IL-10^+^ cells were decreased in the skin lesions in the IRF5 KO mice compared to the WT mice (Figure 3B). These results suggested that augmented Th17 responses and reduced IL-10 expression had a greater impact on the psoriasis-like skin inflammation than the abrogated type I interferon and inflammatory cytokine responses in the IRF5 KO mice, resulting in the exacerbation of clinical symptoms and histochemical changes.

### 2.3. Decreased IL-10 Expression and Increased IL-23 Expression by Dendritic Cells in IRF5 KO Mice

**Figure 4:**
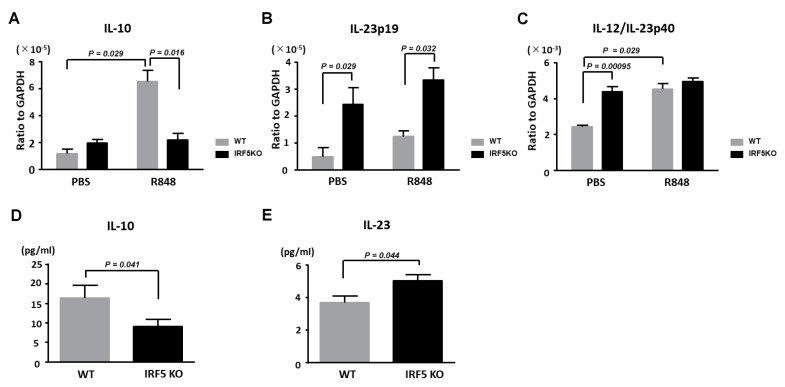
IRF5 deficiency downregulates IL-10 expression and upregulates IL-23 expression by dendritic cells. Dendritic cells (DCs) from the wild-type (WT) or IRF5 knockout (IRF5 KO) mice were stimulated with phosphate-buffered saline (PBS) or 1 µg/mL R848 for 24 h. Messenger RNA levels of IL-10 (**A**), IL-23p19 (**B**) and IL-12/23p40 (**C**) were determined by quantitative RT-PCR. Protein levels of IL-10 in the supernatants after R848 stimulation (**D**) IL-23 in the supernatants without R848 stimulation (**E**) were determined by enzyme-linked immunosorbent assay (ELISA). Data are obtained from duplicate samples from 12 mice in each group. Values are presented as mean the ± SEM of three independent experiments.

IRF5 was mainly expressed in monocytes, macrophages, B cells and DCs [26,27]. Thus, we next examined the IL-10 and IL-23 expression by DCs, a key cell type in the pathogenesis of psoriasis. We stimulated CD11c^+^ DCs from lymph nodes with R848 (TLR7 ligand) and measured IL-10, IL-23p19, and IL-23p40 mRNA expression levels by quantitative reverse-transcription PCR. The augmented IL-10 mRNA expression caused by R848 was abrogated in the IRF5 KO mice (Figure 4A), consistently with the previous report demonstrating that IRF5 is required for the IL-10 production downstream of TLR7 signaling in bone marrow-derived DCs [28]. Although the IL-23 expression in THP-1 cells, a cell line of human monocytes, was reported to be decreased after R848 stimulation when IRF5 was knocked down by CRISPR technology [29], we found that increased IL-23p19 and IL-23p40 mRNA expression occurred without R848 stimulation in DCs from the IRF5 KO mice (Figure 4B,C). In addition, the IL-23p19 mRNA expression levels were higher in DCs from the IRF5 KO mice even after R848 stimulation (Figure 4B,C). We also measured IL-10 and IL-23 protein levels in the supernatants. Consistently with the results of quantitative reverse-transcription PCR, the IL-10 protein levels in the supernatants of DCs after R848 stimulation were also significantly lower in the IRF5 KO mice than in the WT mice (Figure 4D). We also detected that IL-23 protein levels were higher in the supernatants of DCs without R848 stimulation from the IRF5 KO mice than those from the WT mice (Figure 4E). Thus, the lack of IL-10 expression under the TLR7 signaling and the constitutive upregulation of IL-23 in DCs could result in the exacerbation of imiquimod-induced psoriasis-like skin inflammation.

### 2.4. Upregulated IRF-4 Expression in Dendritic Cells from IRF5 KO Mice

**Figure 5:**
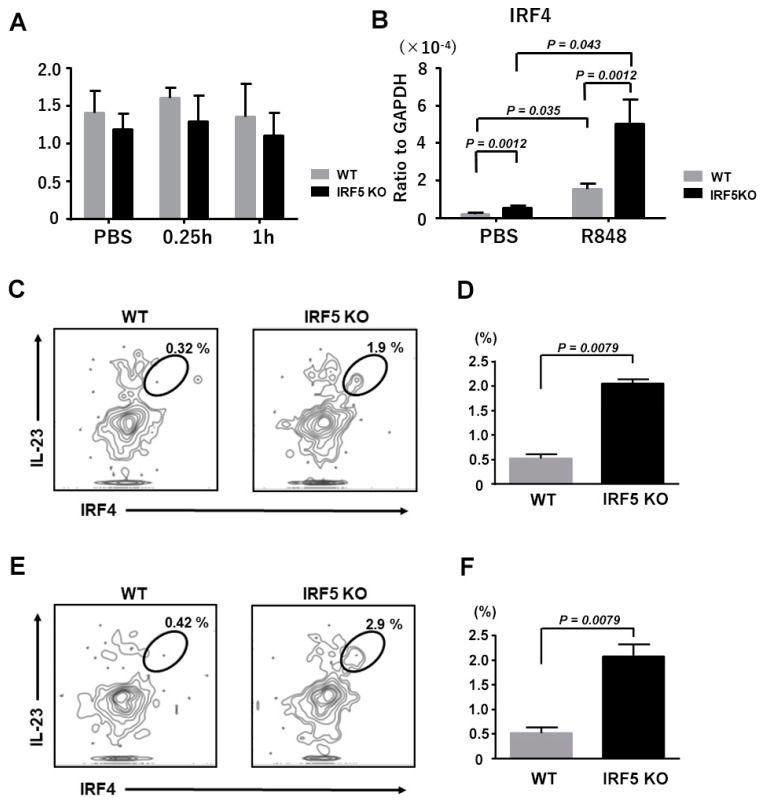
IRF4 expression levels were upregulated in dendritic cells from the IRF5-deficient mice. (**A**) Phosphorylated NF-κB p65 expression in DCs stimulated with PBS or 1 μg/mL R848 for a quarter of an hour or one hour was measured. (**B**) IRF4 mRNA expression levels in DCs before and after stimulation with 1 μg/mL R848 for 24 h were determined by quantitative RT-PCR. Data are obtained from duplicate samples from 12 mice in each group. Values are presented as the mean ± SEM of three independent experiments. (**C**,**E**) Representative flowcytometryplots of IRF4^+^IL-23^+^ DCs before (**C**) and after (**E**) stimulation with 1 μg/mL R848 for 3 h. (**D**,**F**) The frequency of IRF4^+^IL-23^+^ DCs before (**D**) and after (**F**) stimulation with 1 μg/mL R848 for 3 h. Data are obtained from 5 mice in each group. Values are presented as mean the ± SEM of two independent experiments.

Based on the results above, we hypothesized that some signaling pathways leading to IL-23 expression was constitutively activated in DCs from the IRF5 KO mice. We first focused on nuclear factor (NF)-κB pathways, because IRF5 and NF-κB cooperate to provide IL-23 in R848-stimulated THP-1 cells [29]. We measured phosphorylated NF-κB p65 expression in the cell lysates of DCs before and after R848 stimulation, only to find that the expression levels were comparable between the IRF5 KO mice and the WT mice (Figure 5A). We next focused on IRF4, as CD11c^+^ DCs from IRF4 siRNA-treated mice exhibited significant a constitutive reduction of the IL-23p40 expression [30], suggesting that IRF4 can also be involved in the IL-23 expression in DCs. IRF4 expression was upregulated in DCs from IRF5 KO mice both in steady state and under stimulation with R848 (Figure 5B). In addition, the IRF4^+^ IL-23-producing DCs were increased in IRF5KO mice (Figure 5C,D). Thus, the IRF4 expression was augmented in DCs from the IRF5 KO mice, possibly resulting in an increased IL-23 expression.

## 3. Discussion

This study demonstrated whether and how the congenital deficiency of IRF5 was involved in the immunological development of psoriasis using an imiquimod-induced psoriasis-like skin inflammation. IRF5 signaling has been known to induce various inflammatory cytokines and immune responses and IRF5 deficiency ameliorated the symptoms of several systemic lupus erythematosus mouse models, such as MRL/lpr and pristane models and K/BxN serum-transfer arthritis in one of the rheumatoid arthritis mouse models [31,32,33,34]. On the contrary, in the present study, IRF5 deficiency unexpectedly exacerbated psoriasis-like skin inflammation.

Whereas IFN-α and inflammatory cytokines, such as TNF-α and IL-6, were decreased, Th17 cytokines such as IL-17A, IL-23p19, IL-23p40 and IL-22 were significantly higher in the IRF5 KO mice than in the WT mice. IFN-α produced from the activated plasmacytoid DCs plays a crucial role in the early phase of psoriasis, further activating dermal DCs and triggering downstream T cell-mediated adaptive immunity [6], whereas in a imiquimod-induced psoriasis-like skin inflammation model, the signaling via TLR7 was intermittently activated and could directly induce IL-23, TNF-α, and IL-6 expression from DCs [35]. Thus, the downregulation of IFN-α might not have an impact on the inflammation to the same extent as the increase in Th17 cytokines in the IRF5KO mice. Regarding inflammatory cytokines, imiquimod-induced skin inflammation was reported to be ameliorated but not abolished in IL-17 receptor A-deficient mice and that the inflammation was mediated by TNF-α and IL-6, suggesting their importance in the inflammation [36]. Therefore, it is difficult to conclude which are more important for imiquimod-induced skin inflammation, Th17 cytokines or inflammatory cytokines including TNF-α and IL-6. Considering our results, amplified Th17 responses could have a stronger influence on the development of imiquimod-induced skin inflammation than a decrease in TNF-α and IL-6 in IRF5 KO mice. In addition, the decreased IL-10 mRNA expression levels were also detected in the IRF5 KO mice and thought to be associated with the exacerbation of skin inflammation.

We next focused on the IL-10 and IL-23 expression from DCs. As expected, IL-10 expression from DCs under R848 stimulation was diminished in the IRF5 KO mice, consistently with the previous report [28]. To our surprise, but in line with the results of in vivo experiments, IL-23p19 and IL-23p40 expression were constitutively upregulated in DCs from the IRF5 KO mice. On the other hand, it was previously reported that IRF5 knockdown in THP-1 cells resulted in a declined IL-23 expression under R848 stimulation [29]. The cellular type is different between our study and the previous study, but the biggest difference would be whether IRF5 was knocked down congenitally or not. Thus, we hypothesized that some signaling pathways or molecules concerning IL-23 production were upregulated in DCs from the IRF5 KO mice. Although the NF-κB pathways were not activated in IRF5 KO mice, we found that the IRF4 expression was increased in DCs from the IRF5 KO mice. IRF4 has been reported to act as an antagonist of IRF5 and the knockdown of IRF4 resulted in the elevation of IRF5 expression in Epstein–Barr virus-transformed cells [37]. Recently, IRF4 has been confirmed as a target gene of IRF5 and vice versa [38], suggesting that congenital IRF5 deficiency can affect IRF4 expression levels as shown in our results.

There have been several reports describing that IRF4 is involved in the IL-23 expression in DCs. For example, CD11c^+^ DCs from the IRF4 siRNA-treated mice showed significantly decreased inflammatory cytokines including IL-23, resulting in ameliorating experimental autoimmune encephalomyelitis in IRF4 siRNA-treated mice [20]. Murine lung and gut propria contained IRF4-dependent CD24^+^CD11b^+^ DCs that secreted IL-23 and the loss of such DCs abrogated Th17 responses both in steady state and after *Aspergillus* challenge [39]. Additionally, in human CD1c^+^ DCs, the equivalent of murine CD24^+^CD11b^+^ DCs also expressed IRF4 and secreted IL-23 [39]. Moreover, in a murine model of experimental stroke, IRF4^+^ conventional type 2 DCs rapidly infiltrated into the ischemic brain and became the major source of IL-23, followed by IL-17-dependent secondary tissue damage [40]. Consistently with these previous reports, IRF4^+^ IL-23-producing DCs were increased in the lymph nodes of IRF5 KO mice both in steady state and after R848 stimulation. In addition, IRF4 plays an important role in the migration or survival of migratory dermal conventional type 2 DCs in the draining lymph nodes [41]. Thus, the increased IRF4 expression might be associated with the increased number of MHC class II^+^ cells in lesional skin in our study. Consistently, in lesional skin of psoriasis, IRF4 is expressed in dermal infiltrating cells including dermal conventional type 2 DCs and the expression levels are elevated compared to healthy skin [42,43,44].

In conclusion, our results demonstrate that both IRF5 and IRF4 may have an important role in the pathophysiology of psoriasis. Congenital IRF5 deficiency induces the upregulation of IRF4 in DCs followed by an augmented IL-23 production from DCs, resulting in the amplification of Th17 responses and the exacerbation of imiquimod-induced psoriasis-like skin inflammation. The regulation of IRF4 or IRF5 expression may be a novel therapeutic approach to psoriasis.

## 4. Materials and Methods

### 4.1. Mice

The IRF5 KO mice with C57BL/6 background described previously [20] were kindly gifted from Dr. T. Taniguchi (Department of Molecular Immunology, Institute of Industrial Science, The University of Tokyo). Mice were all 8–12 weeks old for all experiments. Age- and sex- matched WT C57BL/6 mice (CLEA Japan, Tokyo, Japan) were used as the controls for the IRF5 KO mice. All mice were maintained under a 12 h light/12 h dark cycle in a specific pathogen-free barrier facility. They were healthy, fertile and did not display evidence of infection or disease. All the studies and procedures were approved by the Committee on Animal Experimentation of the University of Tokyo Graduate School of Medicine on February 15^th^, 2018 (Project code: P17-103).

### 4.2. Induction of Psoriasiform Skin Inflammation by Imiquimod

A daily topical dose of 62.5 mg of commercially available imiquimod cream (5%) (Beselna Cream; Mochida Pharmaceuticals, Tokyo, Japan) was applied to the shaved back skin and ears of mice for 5 consecutive days (days 0–4). Disease severity was assessed by using a scoring system based on the clinical Psoriasis Area and Severity Index. To be precise, the erythema, scaling and thickening were scored independently on a scale from 0 to 4 (0, none; 1, slight; 2, moderate; 3, marked; 4, very marked), and the cumulative score was used as a total score (scale 0–12).

### 4.3. Histological and Immunohistochemical Analysis

The samples of imiquimod-applied mouse back skin were harvested. They were formalin-fixed and stained with hematoxylin and eosin. For immunohistochemistry, the mouse skin was embedded in an optimal cutting compound, snap-frozen in liquid nitrogen and stored at −80 °C. Cryosections were fixed with cold acetone for 5 min and incubated overnight at 4 °C with anti-mouse MHC class II antibody (1/50 dilution, Abcam, Cambridge, UK) and anti-mouse CD3 antibody (1/100 dilution, Abcam). Tissues were subsequently stained with an avidin-biotin peroxidase complex using a Vector ABC staining kit (Vector Laboratories, Burlingame, CA). Diaminobenzidine was used for visualizing the staining, and counterstaining with Mayer hematoxylin was performed according to the manufacturers’ instructions. Stained cells were counted in 10 random grids under high original magnification (×400) power fields of a light microscope. Each section was examined independently by two investigators in a blinded manner. In some experiments, 5 μm-thick tissue sections from freshly frozen samples were stained with fluorescein isothiocyanate (FITC)-conjugated IL-17A (BioLegend, San Diego, CA, USA) and FITC-conjugated IFN-α (Biorbyt, Cambridge, UK), or FITC-conjugated IL-10 (BioLegend).

### 4.4. RNA Isolation and Quantitative Reverse-Transcription PCR Analysis

RNA was obtained from the back skin with an RNeasy Fibrous Tissue Mini Kit (QIAGEN, Valencia, CA, USA). Complementary DNA was synthesized using a Rever Tra Ace qPCR RT Master Mix (Toyobo, Osaka, Japan). The quantitative reverse-transcription PCR assay was carried out using SYBR Green PCR Master Mix (Toyobo) on an ABI Prism 7000 sequence detection system (Life Technologies, Carlsbad, CA, USA). The mRNA levels were normalized to those of the glyceraldehyde-3-phosphate dehydrogenase (GAPDH) gene. The relative change in the levels of genes of interest was determined by the 2^–ΔΔCT^. Primers for mouse IL-17A, IL-23p19, IL-12/23p40, IL-22, IFN-γ, IL-12p35, IL-1β, IL-36α, IL-36γ, IFN-γ, TNF-α, IL-6, iNOS, IL-10, IRF4 and GAPDH were as follows: IL-17A forward, 5′-ATC CCT CAA AGC TCA GCG TGT C-3′ and reverse, 5′-GGG TCT TCA TTG CGG TGG AGA G-3′; IL-23p19 forward, 5′-TGT GCC TAG GAC TAG CAG TCC TGA-3′ and reverse, 5′-TTG GCG GAT CCT TTG CAA GCA GAA-3′; IL-12/23p40 forward, 5′-CTC ACA TCT GCT C-3′ and reverse, 5′-AAT TTG GTG CTT CAC A-3′;IL-22 forward, 5′-AGC TTG AGG TGT CCA ACT TC-3′ and reverse, 5′-GGT AGC ACT CAT CCT TAG CAC TG-3′; IFN-γ forward, 5′-AGC AAC AGC AAG GCG AAA A-3′ and reverse, 5′-CTG GAC CTG TGG GTT GA-3′; IL-12p35 forward, 5′-ACT CTG CGC CAG AAA CCT C-3′ and reverse, 5′-CAC CCT GTT GAT GGT CAC GAC-3′; IL-1β forward, 5′-CTC CAT GAG CTT TGT ACA AGG-3′ and reverse, 5′-TGC TGA TGT ACC AGT TGG GG-3′; IL-36α forward, 5′-TGC CCA CTC ATT CTG ACC CA-3′ and reverse, 5′-GTG CCA CAG AGC AAT GTG TC-3′; IL-36γ forward, 5′-ATG GAC ACC CTA CTT TGC TG-3′ and reverse, 5′-TGT CCG GGT GTG GTA AAA CA-3′; IFN-γ forward, 5′-GTC CTG GCA CAG ATG AGG A-3′ and reverse, 5′-ACC TTC TCC AGG GGG AAT C-3′; TNF-α forward, 5′-ACC CTC ACA CTC AGA TCA TCT TC-3′ and reverse, 5′-TGG TGG TTT GCT ACG T-3′; IL-6 forward, 5′-GAT GGA TGC TAC CAA ACT GGA T-3′ and reverse, 5′-CCA GGT AGC TAT GGT ACT CCA GA-3′; iNOS forward, 5′-CGA AAC GCT TCA CTT CCA A-3′ and reverse, 5′-TGA GCC TAT ATT GCT GTG GCT-3′; IL-10 forward, 5′-TTT GAA TTC CCT GGG TGA GAA-3′ and reverse, 5′-ACA GGG GAG AAA TCG ATG ACA-3′; IRF4 forward, 5′-GCC CAA CAA GCT AGA AAG-3′ and reverse, 5′-TCT CTG AGG GTC TGG AAA CT-3′; GAPDH forward, 5′-CGT GTT CCT ACC CCC AAT GT-3′ and reverse, 5′-TGT CAT ACT TGG CAG GTT TCT-3′.

### 4.5. Cell Isolations and DC Cultures

CD11c^+^ DCs from lymph nodes were selected with anti-CD11c beads according to the protocol of CD11 MicroBeads (Miltenyi Biotech, Bergisch Gladbach, Germany) and cultured in a Roswell Park Memorial Institute (RPMI) medium 1640 with 10% fetal bovine serum. DCs (1.0 × 10^5^ cells per 200 μL well) were stimulated with 1 μg/mL of R848 (InvivoGen, San Diego, CA, USA) for 24 h and the total RNA was then isolated using Trizol (Invitrogen, Waltham, MA, USA). Then, the mRNA expression levels were determined as mentioned above. The supernatants were also harvested and the IL-23 and IL-10 protein levels were quantified using a mouse IL-23 Quantikine ELISA Kit (R&D Systems, Minneapolis, MN, USA) and a mouse IL-10 Quantikine ELISA Kit (R&D Systems), according to the manufacturer’s instructions. In some experiments, the DCs were stimulated with 1 μg/mL of R848 for 15 min or 1 h and the phosphorylated NF-κB p65 expression in DCs was analyzed with a NFκB p65 ELISA kit (Enzo Life Sciences, Tokyo, Japan) according to the manufacturer’s instructions. These assays employed the quantitative sandwich enzyme immunoassay technique. Optical densities were measured at 450 nm using a Bio-Rad Model 550 microplate reader (Bio-Rad Laboratories, Hercules, CA, USA).

### 4.6. Intracellular Flow Cytometry

DCs (1.0 × 10^5^ cells per 200 μL well) were stimulated with 1 μg/mL of R848 and 5 μg/mL monensin (Invitrogen) for 4 h. Isolated DCs were stained with allophycocyanin (APC) anti-CD11c antibody (BioLegend) and FITC anti-MHC class II antibody (eBioscience, Waltham, MA, USA). After surface staining, DCs were permeabilized using a fixation/permeabilization buffer (eBioscience) and PE-Cy7 anti-IRF4 antibody (BioLegend), PE anti-IL-23 antibody (R&D Systems), or isotype-matched control antibody (BioLegend) was added. Cells were washed and analyzed on a FACScan flow cytometer.

### 4.7. Statistical Analysis

The data obtained were presented as the mean ± SEM. Statistical analysis was carried out with one-way ANOVA with Bonferroni post hoc tests for multiple group comparisons and the two-tailed unpaired t-test for two group comparisons. For comparing two group values that did not follow Gaussian distribution, the two-tailed Mann-Whitney u test was used. Values of *p* < 0.05 were considered to represent a significant difference.

## Figures and Tables

**Table 1:** The summary of the results of the messenger RNA expression levels in the skin lesions in IRF5 knockout (IRF5 KO) mice compared to wild-type mice.

**Cytokines Increased in IRF5 KO Mice**	IL-17A, IL-23p19, IL-12/23p40, IL-22, IFN-γ, IL-36γ
**Cytokines Decreased in IRF5 KO Mice**	IFN-α, TNF-α, IL-6, iNOS, IL-10, IL-12p35
**Cytokines not Changed in IRF5 KO Mice**	IL-1β, IL-36α
